# The Evolving Roles of Macrophages in Organ Transplantation

**DOI:** 10.1155/2019/5763430

**Published:** 2019-04-24

**Authors:** Junhui Li, Cai Li, Quan Zhuang, Bo Peng, Yi Zhu, Qifa Ye, Yingzi Ming

**Affiliations:** ^1^Organ Transplantation Center, 3rd Xiangya Hospital, Central South University, Changsha, Hunan 410013, China; ^2^Engineering and Technology Research Center for Transplantation Medicine of the National Ministry of Health, 3rd Xiangya Hospital, Central South University, Changsha, Hunan 410013, China

## Abstract

Organ transplantation is a life-saving strategy for patients with end-stage organ failure. Over the past few decades, organ transplantation has achieved an excellent success in short-term survival but only a marginal improvement in long-term graft outcomes. The pathophysiology of graft loss is multifactorial and remains incompletely defined. However, emerging evidence suggests macrophages as crucial mediators of acute and chronic allograft immunopathology. In this process, macrophage-mediated mobilization of first-line defenses, particularly phagocytosis and the release of acute inflammatory mediators, is important, but macrophages also launch adaptive alloimmune reactions against grafts through antigen processing and presentation, as well as providing costimulation. Additionally, crosstalk with other immune cells and graft endothelial cells causes tissue damage or fibrosis in transplanted organs, contributing to graft loss or tolerance resistance. However, some macrophages function as regulatory cells that are capable of suppressing allogeneic T cells, inhibiting DC maturation, inducing the differentiation of Tregs, and subsequently promoting transplant tolerance. This functional diversity of macrophages in organ transplantation is consistent with their heterogeneity. Although our knowledge of the detrimental or beneficial effects of macrophages on transplants has exponentially increased, the exact mechanisms controlling macrophage functions are not yet completely understood. Here, we review recent advances in our understanding of the multifaceted nature of macrophages, focusing on their evolving roles in organ transplantation and the mechanisms involved in their activation and function in allograft transplantation. We also discuss potential therapeutic options and opportunities to target macrophage to improve the outcomes of transplant recipients.

## 1. Introduction

Organ transplantation has been a life-saving strategy for patients with end-stage organ failure. However, allografts have been reported to induce alloimmunity and result in graft loss without immunosuppression [1]. In essence, this complicated immunopathology is a multifactorial process involving adaptive and innate immunity. Following numerous advances in immunosuppressants targeting adaptive immune cells, short-term graft survival has achieved tremendous success, with one-year total survival rate exceeding 80% [2]. Nonetheless, this huge success has not translated into long-term benefits, as most grafts are lost over time [3, 4]. Thus, current immunotherapeutic strategies targeting adaptive immune cells display limited effectiveness in promoting long-term graft survival, and researchers have expressed doubt regarding the exclusive role of adaptive immune cells in mediating allograft rejection. Actually, emerging studies suggest that both adaptive immune cells and innate immune cells participate in transplant rejection [5–7]. Moreover, graft rejection in immunosuppressed patients emphasizes the key role of innate immune cells in graft rejection, including dendritic cells (DCs) [8], macrophages [9, 10], natural killer (NK) cells [11], and mast cells [12].

Tissue macrophages comprise tissue-resident macrophages and recruited monocyte-derived macrophages, both of which play key roles in innate immunity. Macrophages are crucial mediators of transplant immunopathology. Although macrophages mobilize first-line defenses against pathogens and launch adaptive immunity in the form of antigen-presenting cells (APCs) to protect against infection, macrophages also attack allografts as a foreign entity and contribute to graft loss through a similar mechanism [13, 14]. In contrast, in certain preclinical studies and clinical trials, some macrophage subsets have been reported to function as regulatory cells, and adoptive transfer of these macrophages significantly prolongs graft survival. Thus, macrophages have diverse and highly complex roles within allografts, and their various impacts on transplant outcomes reflect the need to reassess the roles of various types of macrophages in organ transplantation.

Emerging evidence reveals the involvement of macrophages in organ transplantation. In mouse transplant models, monocyte cells are capable of recognizing allogeneic nonself antigens independent of lymphoid cells and play crucial roles in triggering alloimmunity and graft rejection [6, 15, 16]. Furthermore, licensed macrophages reject allograft cells via phagocytosis in presensitized models [14]. Deletion or inhibition of macrophages can attenuate graft injury and prolong graft survival [17]. Consistent with the results from animal studies, clinical studies also identified a positive correlation between macrophages and allotransplant rejection [18–23]. Additionally, antibody-mediated rejection is characterized by the infiltration of monocytes and macrophages [24, 25]. Macrophage accumulation is even considered as a predictor of death-censored graft failure and a potential diagnostic marker of transplant rejection [26]. Additionally, in heart transplant recipients, macrophages are recognized as one of histological diagnostic criteria [24, 27]. Furthermore, recent studies identified a subset of macrophages that suppress allogeneic T cell proliferation and inhibit DC maturation [28, 29]. Meanwhile, adoptive transfer of these macrophages promotes graft survival and minimizes immunosuppression [28, 30, 31]. The bulk of evidence has kindled great interest in the role of innate immune cells in transplantation, which leads to advances in our understanding of the roles of macrophages in organ transplant.

In this review, we provide a comprehensive update on recent advances in determining the roles of macrophages in organ transplantation, including rejection and transplant tolerance. We also discuss the underlying mechanisms of macrophage activation and function within allografts. We conclude by highlighting a new perspective on therapeutic strategies, as macrophage subsets can potentially be manipulated to control allograft rejection and even contribute to immune tolerance.

## 2. Macrophage Immunobiology

Macrophages are phagocytic innate immune cells that are crucial components in host defense and tissue homeostasis mechanisms. Macrophages in the tissue comprise tissue-resident macrophages and recruited monocyte-derived macrophages. Fate-mapping studies indicated that macrophages are mainly derived from embryonic progenitors in steady-state tissues rather than adult hematopoietic progenitors. However, macrophage fate depends on the organ type, and the relative contribution is altered in different settings. Notably, macrophages are extremely dynamic and highly plastic, as a variety of environmental stimuli alter either the activation status or function of macrophages. Epigenetic and transcriptomic analyses revealed a critical role for the tissue microenvironment in shaping the tissue macrophage enhancer landscape and determining cell identity [32, 33]. Tissue macrophages, with many tissue-specific functions, are shaped by the surrounding microenvironment and in turn are responsible for maintaining tissue homeostasis [34]. In addition, macrophages originating from different tissues show distinct phenotypes and functions, even under the same polarization conditions [35, 36]. Although transcriptional regulation and epigenetic modifications of macrophage subsets are reasonably well studied, researchers have not clearly determined whether humans and mice exhibit the same macrophage populations and functions.

Traditionally, based on stimulation in vitro, macrophages are oversimplified as M1 (stimulated with interferon gamma/lipopolysaccharide (IFN-*γ*/LPS)) and M2 macrophages (stimulated with interleukin-4/interleukin-13 (IL-4/IL-13)) [37–39]. Although this categorization ignores the diversity and complexity of tissue macrophages in vivo, no widely accepted refined classification exists, and we will continue to use this approach in this review.

**Figure 1:**
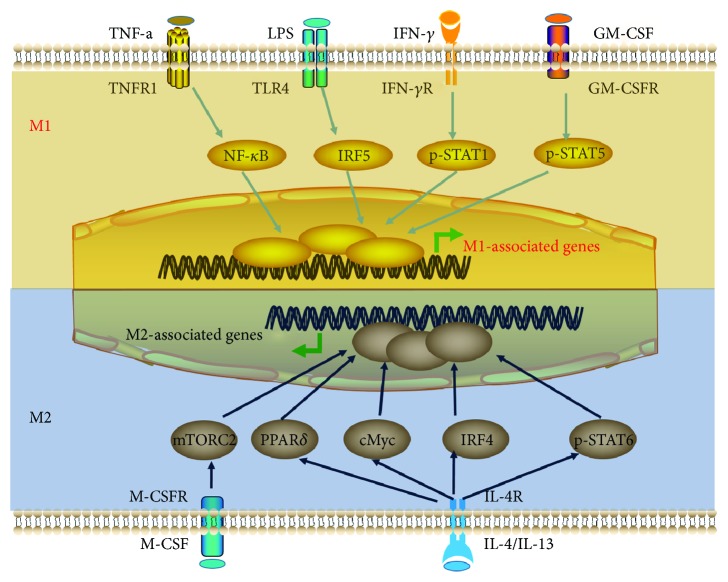
Mechanisms underlying macrophage polarization. The major signaling pathways contributing to the generation of M1 and M2 subsets are outlined. M1 macrophages are polarized following stimulation with IFN-*γ*, LPS, TNF-*α*, and GM-CSF and engagement of TLR4. A predominance of NF-*κ*B, IRF5, STAT1, and STAT5 activation promotes M1 macrophage polarization. In contrast, M2 macrophages are generated following stimulation with IL-4/IL-13. A predominance of STAT6 activation results in enhanced M2 macrophage polarization. PPAR*δ* controls distinct aspects of M2 macrophage activation, and mTORC2 is involved in M2 polarization by regulating glucose metabolism. IL-4–induced c-Myc activation participates in promoting the expression of a subset of M2-associated genes. IL-4 also induces the expression of the M2-polarizing factor IRF4 to inhibit IRF5-mediated M1 polarization. M-CSF promotes M2 polarization through mTORC2 activation, while GM-CSF induces M1 polarization. IFN-*γ*: interferon-*γ*; LPS: lipopolysaccharide; TNF-*α*: tumor necrosis factor-*α*; GM-CSF: granulocyte-macrophage colony-stimulating factor; TLR4: Toll-like receptor 4; NF-*κ*B: nuclear factor-kappa B; IRF5: interferon regulatory factor 5; STAT1: signal transducer and activator of transcription 1; STAT5: signal transducer and activator of transcription 5; IL-4: interleukin 4; IL-13: interleukin 13; STAT6: signal transducer and activator of transcription 6; PPAR*δ*: peroxisome proliferator-activated receptor *δ*; mTORC2: mammalian target of rapamycin complex 2; M-CSF: macrophage colony-stimulating factor.

M1 macrophages, also called “classically activated macrophages,” tend to be proinflammatory cells that secrete proinflammatory cytokines, such as IL-1, IL-6, tumor necrosis factor-*α* (TNF-*α*), and IL-23. M1 macrophages are also capable of eradicating bacterial, fungal, or viral infections. They are polarized following stimulation with IFN-*γ*, LPS, TNF-*α*, and granulocyte-macrophage colony-stimulating factor (GM-CSF) and the engagement of Toll-like receptors (TLR) by microbial products or DAMPs (Figure 1). This subset is characterized by the high expression of inducible nitric oxide synthase (iNOS). Although M1 macrophages contribute to anti-infection responses, their sustained activation, particularly under sterile inflammatory conditions, will lead to tissue injury.

M2 macrophages, which are also described as “alternative activated macrophages,” possess anti-inflammatory functions and are capable of facilitating wound healing, angiogenesis, phagocytosis, fibrosis, and the resolution of inflammation. The polarization of this macrophage subset is induced by IL-4/IL-13. These cytokines activate the JAK-STAT6 pathway, driving the transcription of M2-associated genes, such as *Arg1*, *Mrc1*, *Retnla*, and *Chil3* (Figure 1). High expression of numerous growth factors, including platelet-derived growth factor (PDGF), insulin-like growth factor-1 (IGF-1), and vascular endothelial growth factors (VEGF-*α*), is one of the distinct characters of M2 macrophages. Additionally, M2 macrophages also express mannose receptor 1 (also called CD206), arginase-1 (Arg1), and transforming growth factor beta 1 (TGF-*β*1) at high levels.

## 3. Features of Donor and Graft-Infiltrating Recipient Macrophages

Based on their origins, the macrophages identified in solid organ transplants are divided into donor-resident macrophages, which are transferred to the recipient at the time of transplantation, and recipient monocytes that are recruited into the transplanted organ.

### 3.1. Donor Macrophages

Donor macrophages, which are present in the organ before transplantation, are donor organ tissue-resident macrophages. In the steady state, tissue-resident macrophages are normal components of the tissue stroma, forming part of the first line of defense against invading pathogens. Meanwhile, tissue-resident macrophages are responsible for maintaining tissue homeostasis by phagocytosing necrotic cells and inhibiting inflammatory responses to innocuous stimuli.

Tissue-resident macrophages primarily originate from the yolk sac, the fetal liver, or both tissues; however, some of these cells will be replaced by monocytes derived from adult bone marrow. The relative contributions of these tissue macrophages vary among organ types. For example, kidney-resident macrophages are derived from both yolk sac and hematopoietic progenitors; tissue macrophages in the lung and liver, which are referred to as alveolar macrophages and Kupffer cells, respectively, are mainly derived from the fetal liver and monocytes. Tissue macrophages in the intestine or heart are mainly replaced by adult bone marrow–derived monocytes [40].

Phenotypically, under steady-state conditions, murine tissue-resident macrophages are primarily characterized by the expression of CD11b, F4/80, CD64, CD68, and MerTK and low levels of MHC-II on the cell surface. However, monocyte-derived macrophages are characterized by CD11b, CD209, CD64, and MerTK expression on the cell surface. In humans, tissue-resident macrophages are phenotypically distinguishable and express CD11b, CD64, CD163, CD14, factor XIIIA, and LYVE-1 on the cell surface, while human monocyte-derived macrophages are characterized by CD11b, CD209, CD14, and factor XIIIA expression on the cell surface.

Functionally, under steady-state conditions, tissue-resident macrophages are capable of maintaining peripheral homeostasis by inhibiting T cell activation and proliferation. Brain microglia [41], lung alveolar macrophages [42], Kupffer cells in the liver [43], and lamina propria macrophages [44] exhibit this suppressive function. Additionally, tissue-resident macrophages are thought to be related to the immunogenicity of allograft, as either differences in the origin or subset distribution of the macrophages in the donor tissue may at least partially correlate with the differences in immunogenicity of the transplanted organ.

### 3.2. Recipient Macrophages

In the graft, IRI following organ harvest and vascular anastomosis triggers the mobilization of mononuclear phagocytes in bone marrow and allograft infiltration [45]. Monocytes and their precursors are recruited from the blood, bone marrow, and spleen to the grafts. The recruitment of these cells is mediated by chemoattractants such as monocyte chemoattractant protein-1 (MCP-1) [46], chemokine (C-C motif) ligand 2 (CCL2) [46], C-C chemokine receptor type 1 (CCR1), chemokine (C-X3-C motif) ligand 1 (CX3CL1) [47], macrophage inflammatory protein-1*α* (MIP-1*α*) [48], and C5aR1 [49, 50]. Subsequently, the cells infiltrate the graft, differentiate into macrophages or DCs, and play a crucial role in determining the outcome of transplantation.

Phenotypically, the origins of graft-infiltrating macrophages are difficult to determine based on their phenotypes; however, cell-tracing strategies using CD45.1/CD45.2 or GFP reporter mice have enabled researchers to distinguish between donor and recipient macrophages [51]. Interestingly, a study of sex-mismatched heart transplant patients revealed that CCR2 facilitates the differentiation between donor macrophages and recipient macrophages within an allograft, as CCR2- macrophages belong to a tissue-resident subset while CCR2+ macrophages are derived from monocytes [52]. Moreover, either CSF1R (CD115) or Gr1 facilitates the differentiation between tissue-resident macrophages and monocyte-derived macrophages, as bone marrow Ly6Chi monocytes or the Gr1+ monocyte subset is selectively recruited into injured organs and differentiates into macrophages [53, 54]. However, these phenotypes vary according to the type of organ and differences exist between mouse and human, which is worthy of further investigation.

Functionally, as first responders, interstitial monocyte-derived cells infiltrate allografts within hours [47]. Monocytes trafficking into IRI are regulated by chemokine receptors, such as CCR2 and CX3C chemokine receptor 1 (CX3CR1), as both CCR2-deficient and CX3CR1-deficient mice showed reduced tissue injury. Egress of these cells into an injured organ was observed within 30 minutes following injury and peaked within 24-48 h [47]. This rapid accumulation of monocyte-derived macrophages is the first step in a conventional reaction that causes further tissue damage.

## 4. Macrophages in Acute Rejection

With the extensive use of immunosuppression, acute rejection is no longer a major hurdle in transplantation; however, the mechanism of acute rejection remains incompletely understood.

Accumulating evidence has suggested a pivotal role for macrophages in acute rejection. Macrophages are involved in IRI, the alloimmune response, and acute graft rejection. Based on human biopsy data, macrophages account for 38–60% of graft-infiltrating leukocytes during acute rejection [55–58]. Consistent with the increased macrophage infiltration, the levels of both chemotactic factors responsible for monocyte recruitment and macrophage colony-stimulating factor (M-CSF) responsible for macrophage proliferation are increased within allografts during the postreperfusion period and acute rejection [59]. Moreover, a whole-genome transcriptome analysis of biopsy samples identified an inflammatory macrophage-associated 3-gene signature that is upregulated during acute rejection and positively correlated with the extent of subclinical graft injury [60]. In contrast, macrophage depletion attenuates graft injury and decreases inflammation in acute rejection models [61, 62]. Macrophage infiltration is even suggested to function as potential biomarker of rejection, graft function, and graft survival. Thus, macrophages represent as an important cell type that mediates acute allograft rejection.

Both IRI and acute rejection models revealed a role for macrophages in tissue injury. Macrophage infiltration occurs rapidly following reperfusion during transplantation. Once in the transplanted organ, activated macrophages produce large amounts of proinflammatory cytokines that damage the tissue, such as IL-1, IL-12, IL-18, IL-6, IL-23, TNF-*α*, and IFN-*γ* [57, 63]. Among these proinflammatory cytokines, TNF-*α* is capable of promoting tubular apoptosis after kidney injury, as neutralization of TNF-*α* leads to decreased renal IRI [64]. Similarly, IL-12 contributes to IRI, as IL-12 deletion exerts a protective effect on IRI [65]. These cytokines promote inflammation and tissue injury through divergent pathways; however, further study is required to determine the association between the cytokine source and cytokine function in mediating IRI and acute graft injury. In addition to cytokine-mediated effects, infiltrated macrophages also lead to allograft tissue damage by producing reactive oxygen species (ROS) and reactive nitrogen species (RNS), which subsequently promote acute rejection [66–69]. Mechanistically, interactions between RNS and ROS promote the generation of cytotoxic peroxynitrites, causing lipid peroxidation. Thus, macrophages are well-equipped effector cells that mediate acute graft injury.

Macrophages are also capable of mediating transplant rejection via activating adaptive alloimmune responses. Both donor macrophages and recipient macrophages, which function as APCs, exert substantial effects on alloimmune responses. Mechanistically, alloimmune T cells are activated following an interaction of their T cell receptors with intact allogeneic MHC molecules on donor cells or donor peptides presented by self-MHC molecules on recipient APCs, or with recipient APCs with intact MHCs transferred from donor APCs. Furthermore, macrophages provide costimulatory signals to facilitate and amplify T cell activation, as costimulatory molecules are expressed at high levels on the macrophage surface. Despite the significant progress in determining the roles of macrophages in tissue injury and organ rejection, the mechanisms by which these macrophages mediate graft loss are not completely understood. Although macrophages play key roles in amplifying T cell-mediated acute rejection, the relative contributions of macrophages and DCs require further study.

## 5. Macrophages in Chronic Rejection

Chronic rejection has become a major cause of allograft loss in the clinical setting. The features of chronic rejection include vascular smooth muscle proliferation, neointima formation, damage and atrophy of parenchymal cells, interstitial inflammation and fibrosis, and, finally, vascular occlusion. Based on findings from emerging studies, macrophages are a critical mediator of chronic rejection. Chronically rejecting allografts are characterized by the interstitial infiltration of macrophages and T cells [70]. Compared with T cell-mediated acute rejection, the macrophages outnumber T cells in the graft in chronic rejection models and human patients [71, 72]. Increased macrophage infiltration within allografts positively correlates with worse graft outcome [19], and macrophage accumulation even functions as a potential diagnostic marker of graft rejection in restricted transplantation models [24, 27]. In addition, impaired macrophage recruitment into grafts mediated by the pharmacological blockade of RhoA/Rock or deletion of RhoA, which is involved in regulating the actin cytoskeleton, inhibits chronic rejection of heart transplants and prolonged graft survival [73, 74]. Similarly, macrophage depletion after transplantation reduces transplant vasculopathy and prolongs graft survival [75–77].

Mechanistically, macrophages likely contribute to chronic rejection by promoting smooth muscle cell proliferation and interstitial fibrosis. These effects are associated with the M2 macrophage subset, which promotes proliferation by secreting a variety of growth factors and exert profibrotic effects. Animal transplant models revealed high levels of M2-associated markers, such as Arg-1, CD206, and Fizz1, on graft-infiltrating macrophages in chronically rejected grafts [75]. Moreover, graft biopsies from patients display a dominant population of M2-polarized macrophages [20, 75]. Furthermore, the accumulation of M2 macrophages correlates with the severity of fibrosis [20, 78]. In contrast, strategic inhibition of graft-infiltrating M2 macrophages with oxidized ATP, whose receptor, P2x7r, is preferentially expressed in M2 macrophages, results in reduced transplant vasculopathy, decreased fibrosis, and prolonged cardiac graft survival [75]. Consistent with these findings, block M2 macrophages by the conditional deletion of mammalian target of rapamycin (mTOR) in macrophages induces long-term graft survival without showing obvious features of chronic rejection [77].

Intriguingly, macrophages also facilitate interstitial fibrosis in chronic allograft injury by undergoing the macrophage-to-myofibroblast transition, which is characterized by the coexpression of macrophage (CD68) and myofibroblast (*α*-smooth muscle actin (*α*-SMA)) markers. The macrophage-to-myofibroblast transition was identified in both patients undergoing chronic allograft rejection and a chronic renal allograft injury model. A fate-mapping study using Lyz2-Cre/Rosa26-Tomato mice revealed that myofibroblasts originating from recipient macrophages account for approximately 50% of the myofibroblasts in renal allografts [79]. Functionally, the accumulation of these cells is associated with allograft function and the severity of interstitial fibrosis. Mechanistically, the macrophage-to-myofibroblast transition is regulated by a Smad3-dependent mechanism, as the deletion of Smad3 impairs the macrophage-to-myofibroblast transition and reduces intestinal fibrosis [79].

Additional roles of macrophage in chronic rejection are also emerging. Macrophages have recently been shown to be involved in alloantibody-mediated chronic rejection, particularly in lung transplantation, although the mechanism remains poorly defined. Lung transplant studies revealed a significant role for donor-derived alveolar macrophages (AMs) in the donor-specific antibody- (DSA-) induced inflammatory cascade, which causes bronchiolar obstruction [80, 81]. Adoptive transfer of allogeneic AMs stimulates lung-restricted humoral and cellular autoimmunity and leads to obstructive airway disease of the transplanted lung [80, 81]. Mechanistically, donor AMs respond to a human leukocyte antigen- (HLA-) specific antibody by secreting proinflammatory cytokines. Furthermore, antigen presentation by AMs requires Zbtb7a, as Zbtb7a deletion failed to induce antibody and T cell responses [81]. Thus, AMs are implicated in anti-HLA-induced lung allograft rejection.

## 6. Macrophages in Graft Tolerance

Although macrophages contribute to allograft rejection through various mechanisms, recent evidence suggested that macrophages are also involved in transplant tolerance, as adoptive transfer of regulatory macrophages (Mregs) induces graft tolerance. Mregs represent a unique macrophage subset that are induced in the presence of M-CSF and IFN-*γ* and is distinguished from M1 and M2 cells [29]. Functionally, these cells are characterized by suppressive activity and anti-inflammatory properties. T cell proliferation is nonspecifically suppressed by Mregs through the preferential elimination of allogeneic T cells [82]. In mice, these functions are mediated by an iNOS-dependent mechanism, as Nos2-deficient Mregs failed to suppress T cell proliferation [83]. In a murine cardiac transplantation model, adoptive transfer of Mregs dramatically improved cardiac allograft survival in nonimmunosuppressed and fully allogeneic recipients; however, the transfer of Nos2-deficient Mregs failed to prolong allograft survival [83].

Another significant issue is that Mregs also function as critical mediators of graft tolerance. Mregs are characterized by the capability to inhibit CD8+T cell immunity and promote CD4+ forkhead box P3 + (Foxp3+) regulatory T cell (Treg) expansion [84]. This type of suppressive macrophage is required to induce graft tolerance, as the depletion of suppressive macrophages in vivo abrogates allograft tolerance, despite a tolerogenic treatment [84]. Mechanistically, simultaneous DC-SIGN engagement by fucosylated ligands and TLR4 pathways promotes the expression of IL-10, which is essential for Mreg-mediated prolongation of allograft survival [84]. Thus, Mregs play a pivotal role in immunological tolerance and have concomitant therapeutic implications in the clinic.

Similar to mouse Mregs, human Mregs are induced following stimulation with M-CSF and IFN-*γ* [29, 84, 85]. Phenotypically, human Mregs are characterized by the expression of DHRS9, which is a unique and robust marker of human Mregs but not mouse Mregs [85]. Human Mregs are capable of suppressing the proliferation and activation of allogeneic T cells, as well as depleting allogeneic T cells [29]. Furthermore, human Mregs rapidly induce TIGIT+Foxp3+-induced Tregs and inhibit DC maturation, promoting allograft acceptance [28]. In the transplant acceptance-inducing cell- (TAIC-) I clinical trial, Mreg-based therapy was administered to 12 patients received kidney transplants from deceased donors. No obvious adverse effects or signs of clinical rejection were observed, which prompted the use of Mreg-targeted therapy as a safe and practical therapeutic approach in renal transplantation [31]. Subsequently, the TAIC-II clinical trial, in which 5 patients underwent living-donor kidney transplantation, confirmed the safety of the Mreg-targeted strategy and suggested that TAICs are highly potent in suppressing alloantigen-specific responses and minimizing immunosuppression [30]. In another clinical trial, an Mreg treatment was administered to 2 patients received living-donor kidney transplantation, resulting in stable renal function with very low-dose tacrolimus monotherapy [29]. Both recipients have survived for more than 6 years after transplantation without showing acute or delayed adverse responses and signs of subclinical rejection. Furthermore, another clinical trial of Mregs is currently in progress, which will recruit 16 living donor kidney transplant recipients (clinicaltrials.gov: NCT02085629).

Clearly, all these data highlight the potential effectiveness of macrophage-based therapies in transplantation; however, further studies are warranted to determine the mechanism by which Mregs mediate transplant tolerance, including the safety and stability of Mregs in vivo and the efficacy of Mreg treatments in a wide and variable population.

## 7. Therapies Targeting Macrophages for Solid Organ Transplant

**Figure 2:**
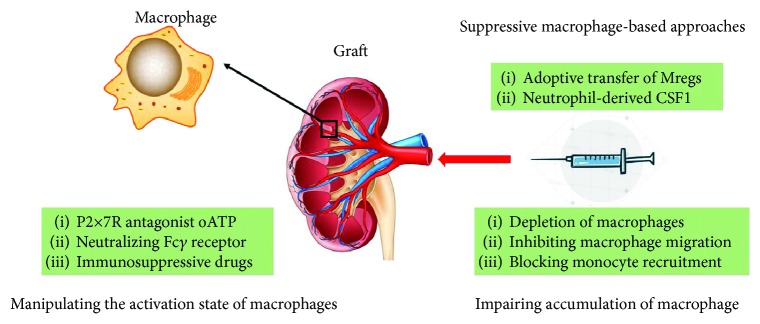
Macrophage-targeted therapy for transplant tolerance. Mregs are generated from bone marrow precursors in rodents or from monocytes in humans. Both the adoptive transfer of Mregs and the expansion of Mregs with neutrophil-derived CSF1 in vivo inhibit the anti-donor T-cell response. Alternatively, strategies that prevent the accumulation of macrophages within allografts, including depletion of macrophages, inhibition of macrophage migration, and blockade of monocyte recruitment, also promote graft survival and contribute to graft acceptance. Strategies suppressing the activation of macrophages with detrimental functions involved in alloimmunity might be an effective therapy, such as inhibiting M2 polarization with the P2x7R antagonist oATP. Moreover, either neutralization of the Fc*γ* receptor or treatment with several immunosuppressive drugs (glucocorticoids, rapamycin inhibitors, and mycophenolate mofetil) exerts suppressive effects on proinflammatory macrophages and prolongs graft survival.

Current studies identified both the beneficial and detrimental effects of macrophages on various solid organ transplant models. The essential roles of macrophages in transplantation have indicated the therapeutic potential of macrophage-targeted treatment in organ transplantation. Thus, the mechanisms underlying macrophage migration, activation, and action might be potential therapeutic targets. In principle, strategies targeting macrophages in an attempt to prolong graft survival and even graft tolerance include preventing macrophage accumulation within the allograft, manipulating their activation, and inducing suppressive macrophages in favor of inducing tolerance (Figure 2).

### 7.1. Preventing the Accumulation of Macrophages in Allografts

Investigations in animal transplant models have shown that appropriately timed depletion of macrophages or inhibition of macrophage migration maintains graft function and prolong allograft survival. A carrageenan treatment, which exerted negligible effects upon T, B, or NK cells, depleted 30–80% macrophages within cardiac allografts and led to a 70% reduction in transplant vasculopathy [76]. Conditional deletion of monocytes and macrophages in CD11b-DTR mice effectively prevented macrophage infiltration in murine renal allografts and resulted in reduced acute rejection-related tissue injury [17]. Furthermore, the impaired macrophage migration induced by conditional deletion of RhoA in macrophages reduced transplant vasculopathy and interstitial fibrosis and improved graft survival [73]. Additionally, blockade of CSF-1/CSF-1R signaling seems to be a potential therapeutic approach to selectively mitigate macrophage accumulation in allografts, as it has been tested in animal models and patients [86]. Furthermore, inhibition of chemokine- and chemokine receptor-mediated chemotaxis also displays therapeutic value in blocking monocyte recruitment, as either a CCR5/CXCR3 antagonist or a neutralizing antibody against CX3CR1 suppressed macrophage infiltration and prolonged graft survival [87]. However, the nonselective inhibition of macrophage accumulation requires further investigation, as these treatments might blunt the patient's ability to control infections and neglect the fact that the function of regulatory macrophages is required for transplant tolerance. More selective approaches that suppress recruitment of macrophages contributing to allograft injury but not those facilitating transplant acceptance are needed.

### 7.2. Manipulating the Activation of Macrophages

Because of the functional diversity of macrophages involved in graft injury and rejection, strategies manipulating their activation may provide therapeutic value in the clinic. Suppression of the activation of macrophages with detrimental functions in alloimmunity might be an effective therapy. In chronic rejection models, the P2x7R antagonist oATP binds to P2x7R, resulting in impaired M2 polarization and reduced transplant vasculopathy [75]. Additionally, a conditional deficiency of mTOR in macrophages inhibits M2 macrophage activation and prevents allograft rejection via the programmed cell death protein 1/programmed cell death 1 ligand (PD-1/PD-L1) coinhibitory pathway and the expansion of Tregs [77]. Blockade of macrophage-mediated proinflammatory responses might also improve graft survival. The proinflammatory activation of graft-infiltrating macrophages was preferentially inhibited by a short-term treatment with high-density lipoprotein (HDL) nanoparticles loaded with rapamycin (mTORi) and reduced cytokine and lactate production upon restimulation with LPS, suggesting that mTORi-HDL as a possible macrophage-targeted therapy for organ transplantation. In cardiac transplantation models, targeting macrophages with mTORi-HDL markedly prolonged graft survival by reducing the number of graft-infiltrating inflammatory Ly6Chi macrophages and increasing the number of Ly6Clo macrophages [88]. Neutralization of the Fc*γ* receptor suppresses antigen presentation capability of monocytes and inhibits proinflammatory macrophages, but promotes the activity of immunoregulatory macrophages by upregulating IL-10 [89]. In addition, several immunosuppressive drugs used in current clinical practice, including glucocorticoids, rapamycin inhibitors, and mycophenolate mofetil, also partially exert their therapeutic effects by suppressing proinflammatory macrophages [10]. Although these methods are useful for manipulating the activation of macrophages, more specific approaches without apparent systemic side effects are required. A potential alternative strategy would be to selectively inhibit proinflammatory and alloreactive macrophages within allografts and enhance the function of suppressive macrophages or to repolarize graft-infiltrating macrophages to exert a regulatory function. These strategies are attractive, but further studies are required before they are translated to clinical applications.

### 7.3. Suppressive Macrophage-Based Approaches

As suppressive macrophages play an essential role in mediating transplant tolerance, the use of suppressive macrophages as therapeutic agents in adoptive transfer displays substantial therapeutic potential in humans. Adoptive transfer of Mregs in animal models produced promising results, as graft survival was dramatically improved. Although clinical trials of Mregs are limited, the results are promising, as both recipients continue to show stable renal function with a very low dose of tacrolimus monotherapy at more than 6 years after adoptive transfer [29]. Indeed, adoptive transfer of suppressive macrophages has several advantages over conventional therapeutic approaches. Suppressive macrophages have the potential to mediate contact-dependent depletion of activated T cells and drive the differentiation of Tregs from naive non-Tregs, thus avoiding the toxicity of general immunosuppressants. Moreover, a suppressive macrophage treatment can reestablish immunological tolerance and exert lasting therapeutic effects on mice. In addition to the adoptive transfer of Mregs, strategies promoting Mreg polarization in vivo via an M-CSF-dependent pathway also represent a novel therapeutic approach for organ transplantation recipients, as neutrophil-derived CSF-1 promotes Mreg polarization and the suppressive functions of graft infiltrating macrophages [90]. Additionally, the induction of Mregs with nanotechnology tools, such as mTORi-HDL, also promotes transplant tolerance, as these macrophages are capable of suppressing alloreactive CD8+ T cell-mediated immunity and enhancing tolerogenic Treg expansion [88]. However, before suppressive macrophage therapy is translated to the clinic, numerous issues must be addressed with human suppressive macrophages, including the efficiency of Mreg induction, stability, and plasticity in vivo.

## 8. Conclusions

Over the past few years, our understanding of the biology of macrophages in homeostasis and disease has significantly improved. Furthermore, findings from preclinical models of solid organ transplantation and clinical studies reveal the involvement of macrophages in determining transplant outcomes and indicate the therapeutic potential of macrophage-targeted approaches in the clinic. However, the challenge is the heterogeneity of macrophages in vivo, as macrophages are capable of differentiating into phenotypically and functionally distinct subsets, depending on the organ types, the local cytokine milieu, and the crosstalk with other immune cells. Moreover, limited strategies exist to selectively manipulate specific macrophage subsets. Thus, a sufficient number of specific makers must be identified to facilitate the selective targeting of graft-infiltrating macrophage subsets. Although extensive animal studies have revealed the potency and efficacy of macrophage-targeted therapies in organ transplantation, the mechanisms have not defined, and most of them remain unexplored in the clinic.

Obviously, a thorough understanding of macrophage biology is the key to translating macrophage- targeted therapy into clinic practice. Notably, the current categorization schemes do not reflect the diversity and complexity of macrophages in vivo; thus, a consensus classification of macrophage subsets and definitions for novel subsets are urgently needed. Fortunately, studies employing genomics, transcriptomics, and proteomics technologies will considerably facilitate the definition of the heterogeneity and diversity of macrophages in humans and will enable the development of selective strategies that target graft-infiltrating macrophages to improve the outcomes of transplant recipients.
